# Framework Materials for Full-Arch Implant-Supported Rehabilitations: A Systematic Review of Clinical Studies

**DOI:** 10.3390/ma14123251

**Published:** 2021-06-12

**Authors:** Francesca Delucchi, Emanuele De Giovanni, Paolo Pesce, Francesco Bagnasco, Francesco Pera, Domenico Baldi, Maria Menini

**Affiliations:** 1 Division of Prosthodontics and Implant Prosthodontics, Department of Surgical Sciences (DISC), University of Genova, 16132 Genova, Italy; francesca.delucchi@edu.unige.it (F.D.); lele9-90@hotmail.it (E.D.G.); paolo.pesce@unige.it (P.P.); fcbagna5@hotmail.it (F.B.); domenico.baldi@unige.it (D.B.); 2 CIR-Dental School, University of Turin, 10126 Turin, Italy; francesco.pera@unito.it

**Keywords:** framework, restorative material, dental implants, full-arch, titanium, carbon-fiber, zirconia, systematic review

## Abstract

The purpose of this systematic review was to investigate the clinical outcomes of frameworks made of different materials in patients with implant-supported full-arch prostheses. A literature search was conducted on MEDLINE, Scopus and Cochrane Library, until the 1st of March 2021, with the following search terms: framework or substructure combined with “dental implants”. The outcomes evaluated were: implant and prosthesis survival, bone resorption, biological and technical complications. The Cochrane Handbook for Systematic Reviews of Interventions was employed to assess the risk of bias in randomized clinical trials. The Newcastle–Ottawa quality assessment scale was used for non-randomized studies. In total, 924 records were evaluated for title and abstract, and 11 studies were included in the review: 4 clinical randomized trials and 7 cohort studies. The framework materials investigated were: gold alloy, titanium, silver-palladium alloy, zirconia and polymers including acrylic resin and carbon-fiber-reinforced composites. High implant and prosthetic cumulative survival rates were recorded by all included studies. Various materials and different fabrication techniques are now available as alternatives to traditional cast metal frameworks, for full-arch implant-supported rehabilitations. Further long-term studies are needed to validate the use of these materials and clarify their specific clinical indications and manufacturing protocols to optimize their clinical outcomes.

## 1. Introduction

Nowadays, implant-supported full-arch rehabilitations can be considered a predictable treatment solution in cases of complete edentulism [1]. However, the long-term success of these procedures depends on a correct treatment plan, taking in due consideration both surgical and prosthodontic aspects of the rehabilitation.

According to a systematic review by Pjetursson, technical complications occur more frequently in implants than in natural tooth-borne prostheses [2]. This underlines the importance of a careful design of the prothesis shape and materials in Implant Prosthodontics.

In particular, when immediate loading protocols are applied, clinicians should implement load control, in order to minimize implant micromovements and obtain an adequate osseointegration [3].

A proper load control depends both on patient-related factors and on prosthesis design. The presence of a stiff substructure, rigidly splinting the implants together, is supposed to provide a good distribution of occlusal stress more evenly to the abutments and implants [4]. This would prevent high levels of compressive stresses and strains on peri-implant bone, which is particularly important right after placement of implants that will be immediately loaded [5].

In addition, Ogawa et al. [6] have shown that the use of a rigid framework can reduce the bending moments of the prosthesis, and this could be translated into a lower incidence of technical complications.

In implant prosthodontics, gold and its alloys and other metal alloys (e.g., Cr-Co, Au-Pd, Ag-Pd) have traditionally been the gold standard materials for frameworks, in order to increase prosthesis stiffness, protecting implants from overloads and reducing the risk of technical complications. In addition, even when prosthodontic space is limited, metal allows the fabrication of a sufficiently rigid framework. In this way, a more aesthetic prosthesis (without pink soft tissue) can be realized, avoiding aggressive bone remodeling, which would be necessary for the accommodation of a full-acrylic prosthesis stiff and thick enough when the prosthodontic volume is limited [7].

Titanium and its alloys, but also Zirconia, were then introduced as alternative materials, in order to fabricate implant frameworks by CAD/CAM technology, with high implant success rates (92.4–100%) [8]. These two materials are both highly biocompatible, as they prevent galvanic corrosion, which is the typical disadvantage of non-noble metal alloys.

However, in a recent in vitro study, Zirconia frameworks showed higher strain concentration compared to titanium ones. In addition, the authors suggested caution with zirconia frameworks, in case of potential risk factors for mechanical complications (e.g., parafunctional habits) [9].

Another clinical study found a 31.25% porcelain chipping/fracture rate after 2–4 years of function.

Titanium provides good rigidity in the face of a higher flexion resistance than other metals. However, it requires special equipment for its manufacturing, due to its high melting point and reactivity.

Finally, technical complications with both CAD/CAM zirconia and titanium frameworks for implant-supported prostheses were previously reported [10,11].

Metal for fixed-prosthesis frameworks entails high costs and process time. Moreover, its adhesive affinity with acrylic resin is not optimal and often causes the chipping of dental aesthetic veneers from the substructure, with consequential patient discomfort. For this reason, possible alternatives are emerging. Recently, there has been an increasing interest in fiber-reinforced composites (FRC), a group of materials combining a polymer matrix and reinforcing fibers, which were tested for the first time in the 1960s, but more extensively developed and clinically approved for dental use during the last 30 years. Modern FRCs are employed where high static and dynamic strength and fracture toughness, especially in relation to weight, are desired features [12].

Glass fibers, because of their aesthetic properties, have been mainly used as reinforcements of resinous prostheses; however, their stiffness and strength might be insufficient in full-arch fixed prostheses, if compared with metal alloys, especially in clinical cases where the prosthodontic space is limited. On the contrary, as reported in an in vitro study by Menini et al., carbon fibers resin composite (CFRC), due to its optimal mechanical properties and biocompatibility, may be used for the fabrication of frameworks for fixed implant-supported restorations, being a viable alternative to the metal ones [7].

Nowadays, a number of different restorative materials are available for the fabrication of implant-supported full-fixed prostheses: both traditional materials and materials recently introduced thanks to new technologies. Since the choice of the appropriate material might be particularly important in determining the success of the rehabilitation, the aim of the present systematic review was to investigate the clinical outcomes of frameworks made of different materials, in patients rehabilitated with implant-supported full-arch prostheses.

## 2. Materials and Methods

The present systematic review was conducted according to guidelines reported in the indications of the Preferred Reporting Items for Systematic Review and Meta-Analysis (PRISMA) [13].

The focused question was: “What are the clinical outcomes of prostheses with different framework materials in patients rehabilitated with implant-supported full-arch rehabilitations?”. The focused question was established according to PICO strategy:

- Population: patients rehabilitated with fixed implant-supported full-arch prostheses in one or both jaws.
- Intervention: fixed full-arch prothesis realized with any type of framework material (or monolithic prosthesis).
- Comparison: fixed full-arch prothesis realized with a different framework material.
- Outcomes: implant and prosthesis survival (iCSR and pCSR), bone resorption, biological and technical complications.

### 2.1. Search Strategy

The following Internet sources were used to search for papers that satisfied the study’s purpose: National Library of Medicine (MEDLINE—PubMed), Scopus and Cochrane Library. The last search was performed on the 1st of March 2021. We used the following search terms to search all databases: framework or substructure combined with “dental implants”. As an example, the search strategy used on MEDLINE was: (framework OR substructure) AND “dental implants” [mesh]. 

All the clinical studies investigating different framework materials in patients rehabilitated with fixed implant-supported full-arch rehabilitations were included if they met the following inclusion criteria:

- Minimum of 10 patients included;
- At least 1-year follow-up since prosthesis delivery;
- The framework material should be clearly indicated;
- Studies including the comparison of at least two different framework materials.

Eligible articles included: case–control studies, cohort studies and randomized clinical trials. Publications that did not report clinical outcomes of implant-supported full-arch rehabilitations realized using different framework materials and that did not meet the above inclusion criteria were excluded. Papers that were not dealing with original clinical cases (e.g., reviews, conference abstracts, personal opinions, technical notes, editorials, etc.) and multiple publications from the same pool of patients (redundant publications) were excluded. Additionally, reports based on questionnaires or interviews (i.e., studies without clinical examination of the patients) were excluded. No restrictions in terms of year or language of publication were applied. No publication status restrictions were imposed. In addition, full-text articles of narrative and systematic reviews dealing with the topic of the present review were obtained. A hand search was performed by screening these reviews and the reference list of all included publications to select potentially relevant additional studies and to improve the sensitivity of the search.

### 2.2. Screening and Selection

Two independent reviewers (F.D. and E.D.G) read the title and abstract of all articles obtained from the electronic search for possible inclusion. The full texts of all studies of possible relevance were then obtained for independent assessment by the reviewers. Disagreements between reviewers were resolved by discussion between the two review authors; if no agreement could be reached, a third author decided (M.M.). Cohen’s k between the two independent reviewers (F.D. and E.D.G.) was 0.91 (almost perfect agreement). In fact, their only dispute related to the eventual inclusion of two articles found on the Scopus database that were finally excluded according to the decision of M.M., because they did not fulfill all the included criteria.

### 2.3. Data Extraction

Data from the studies included in the final selection were extracted by one of the authors using the Microsoft Excel spreadsheet software (Excel 16.4, Microsoft CO, Redmond, WA, USA) (F.D.). The accuracy of data was verified independently by another coauthor (E.D.G). The following data were extracted: title, author, publication year, study design, follow-up period, type of loading, sample size of test and control group (number of patients and implants), framework material used in test and control group, jaw (maxilla or mandible), application and main outcomes.

### 2.4. Quality Assessment

Cochrane Handbook for Systematic Reviews of Interventions was employed for randomized clinical trials and follow-up [14]. The following quality criteria were assessed: sequence generation, allocation concealment, systematic differences in care provided to members of different study groups other than intervention under investigation (performance bias), systematic differences between groups in how outcomes were determined (detection bias), unequal loss of participants from study groups (attrition bias), within-study selective outcome reporting (selective reporting bias), and other potential risks of bias.

The risk of bias of the included cohort studies was assessed using the Newcastle–Ottawa scale (NOS) [15]. Two reviewers (F.D.; E.D.G) independently evaluated the quality of studies based on the following parameters: selection, comparability, and outcome/exposure. A maximum of 4 stars in the selection domain, 2 stars in the comparability domain and 4 stars in the outcome/exposure domain were given. The included studies were qualified as “good”, “fair” and “poor” quality based on the total NOS score they achieved. Studies with a NOS score ≥ 7 were considered good-quality studies.

## 3. Results

### 3.1. Bibliographic Search and Study Selection

**Figure 1:**
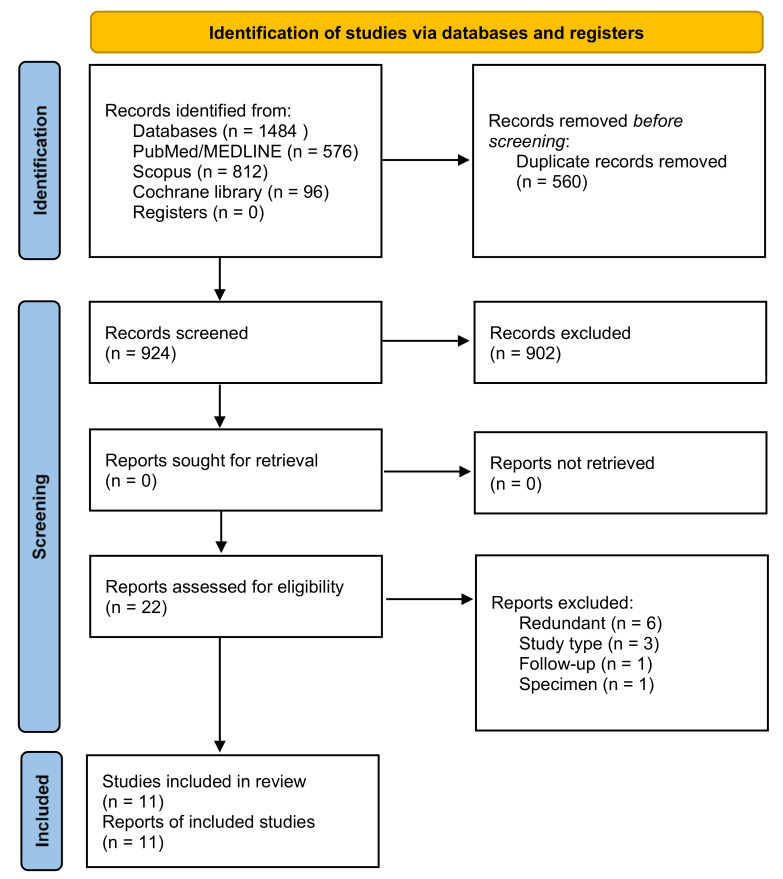
Preferred reporting of systematic reviews and meta-analyses (PRISMA) flow diagram related to bibliographic searching and study selection.

The initial database search yielded a total of 1484 entries, of which 576 were found in PubMed^®^/MEDLINE, 812 in Scopus, and 96 in Cochrane library. A flow chart that depicts the screening process is displayed in Figure 1. After excluding all duplicates, the total number of entries was reduced to 924. A total of 902 articles were excluded after review of the title and abstract. Hence, a full-text examination was conducted for 22 articles.

A total of 11 additional articles were excluded after full-text review and application of the eligibility criteria (the most frequent reason for exclusion being redundancy of publication) [16,17,18,19,20,21,22,23,24,25,26]. The final selection consisted of 11 articles [27,28,29,30,31,32,33,34,35,36,37].

### 3.2. Description of the Included Studies

The 11 included studies are listed in Table 1. They were published between 1999 and 2020. Four studies were conducted in Italy [31,33,34,35]; four in Sweden [27,28], by Ortorp et al. [30,32]; one in UK [29]; one in USA [37], and one in Portugal [36].

All the papers report the clinical outcomes of implant-supported full-arch maxillary and/or mandibular rehabilitations provided with two different framework materials.

Five of the included investigations are prospective cohort studies [27,29,35,36]. Among them, Bergendal and Palmquist [27] and Pera et al. [35] compared the test group with a historical control group. On the other hand, Cannizzaro et al. [34] initially did not design their study in order to compare two different types of framework, but eventually drew conclusions in this sense, after having collected the data. The study by Bergendal and Palmquist [27] is multicentric.

Two studies are retrospective cohort studies [33,37] and three studies are randomized clinical trials (RCT) [28,31,32], including the study by Jemt et al. [28] which is multicentric. The study by Ortorp et al. [30] is a post-trial follow-up (PTFU).

Frameworks made of the following materials were examined: titanium [27,28,30,32,34], gold or other metal alloys [27,28,29,30,31,32,33,34,35,37], zirconia [33,36,37], full-acrylic [31] and PMMA resin [33].

A delayed loading of the implants was applied in four studies [29,30,32,36] and immediate loading in four studies [31,33,34,35]. One study reported that immediate or delayed loading was applied [37], while the type of loading was not reported in the two other studies [27,28].

The most commonly employed material for veneering was acrylic resin, which was reported in seven studies [28,29,30,31,32,34,35]. Two studies used acrylic resin or ceramic as coating materials [33,37]. One study employed zirconia frameworks with feldspathic porcelain veneering, or monolithic zirconia frameworks, coated by porcelain limited to buccal surfaces [36]. One study did not report the veneering material used [27].

Eight of the included studies evaluated screw-retained full-arch implant-supported rehabilitations [29,30,31,33,34,35,36,37]. The other three studies did not report the type of prosthetic retaining [27,28,32].

All the papers included reported high implant and prosthetic cumulative survival rates (in most cases well above 90%). In particular, the minimum and the maximum values (or range of values) of pCSR were found, respectively, by Barootchi et al. 2020 (83.0% ± 11.1%) for the cast metal framework group and by Crespi et al. [31] (100%) for the full-acrylic and metal-acrylic framework group. The minimum and maximum values of iCSR were found, respectively, by Jemt et al. [28] (91.4%) for the laser-welded titanium framework group and by Pera et al. (100%) for the carbon fiber framework group.

All the papers included identified a set of major or minor technical and mechanical complications (e.g., single tooth fracture, framework fracture, dislodgment, chipping, loss of the access chamber composite plug, prosthetic screw loosening, etc.). However, the prevalence of these complications was not considered high by most of the researchers.

The majority of the authors did not find any statistical difference in the two compared groups. The two only exceptions were Cannizzaro et al. [34] and Ortorp et al. [30], who registered a statistically significant higher rate of technical complications in laser-welded titanium compared to traditional cast metal frameworks.

Eight studies [27,28,29,30,31,32,34,35] reported the values of peri-implant marginal bone resorption and statistically significant differences were found only in two studies. In fact, both Pera et al. [35] and Ortorp et al. [30] reported higher bone loss with cast metal frameworks (mean 1 mm and 0.98 mm, respectively) compared to carbon fiber frameworks and laser-welded titanium, respectively. In the majority of the studies, patients had bone changes less than 1 mm; only in Crespi et al. [31] values of bone resorption higher than 1 mm were detected. Bergendal and Palmquist [27] declared that no statistical differences in peri-implant bone resorption were detected, but they did not report numerical data. Cannizzaro et al. [34] only reported an overall value for test and control group (mean 0.69 mm).

Seven studies [27,28,29,30,32,34,37] reported some biological complications, such as soft tissue adverse reactions and/or onset of peri-implant disease. Both Jemt et al. [28] and Bergendal and Palmquist [27] found a significantly higher incidence of biological adverse events in cast metal frameworks, when compared to laser-welded titanium frameworks.

### 3.3. Excluded Studies

Out of 22 papers for which the full text was analyzed, 11 articles were excluded from the systematic review (Appendix A, Table A1), the main reason for exclusion being redundancy of publication.

### 3.4. Quality Assessment

The risk of bias of included studies was assessed using Cochrane Handbook for Systematic Reviews of Interventions (for randomized clinical trials) and the Newcastle–Ottawa scale (NOS) (for cohort studies). Outcomes are reported in Table 2 and Table 3.

In the “adequacy of follow-up of cohorts” section, the authors considered 3% as the maximum rate of subjects lost to follow-up unlikely to introduce bias.

## 4. Discussion

This systematic review focused on the outcomes of comparative clinical studies evaluating full-arch implant-supported fixed prostheses provided with different framework materials.

One of the main limits of the research was the heterogeneity of the studies included. In fact, the different framework material was not the only variable present and the studies differed for several aspects such as: time of implant loading (delayed vs. immediate), number of implants per prosthesis, implant macro- and micro-structure, veneering material, prosthesis connection, type of antagonist, different prosthesis design, different fabrication technique, type of temporary prosthesis, etc. For this reason, a meta-analysis was not feasible.

Seven of the included studies were not randomized cohort studies and their risk of bias was, therefore, assessed using the NOS scale. Four of these studies were considered of good quality (NOS score ≥ 7), and the remaining three studies were considered at high risk of bias (NOS score 6 or 5).

Among the included RCTs, only Jemt et al. [28] had a global low risk of bias. The other three RCTs [30,31,32] were considered at high risk of bias. In fact, they declared a randomization, but the randomization method was not described. In addition, Ortorp et al. [30] divided the test group into two subgroups of treatment, without precisely describing how this procedure was performed. The results of quality assessment demonstrate the need for further research on the topic with a more rigorous methodological design.

Despite the wide diversity of rehabilitative solutions described in the present research, all the selected studies confirm that full-arch implant-supported fixed dental prostheses can be considered a successful treatment option for edentulism, with an implant survival rate well above 90%, with all the studies reporting a minimum follow-up of 1 year up to a 15-year follow-up.

Metal-acrylic resin prosthesis is the traditional treatment protocol for full-arch implant-supported rehabilitations, with high performances, and ease of repair in case of damage of the veneering material. Because of their shock absorption potential, more resilient veneering materials, such as acrylic or composite resin, have been suggested for coating rigid metal frameworks in order to dampen occlusal loads [38]. An in vitro study by Menini et al. [39] has demonstrated that composite and acrylic resin absorb shock from occlusal forces significantly better than ceramics and zirconia, thus reducing loads at the bone–implant interface. This shock absorbing effect coupled with the rigidity of a stiff framework, which is able to evenly distribute loads at the supporting implants, are considered the best option by the authors in order to control occlusal loads.

However, the high costs of traditional gold alloys have led to an increasing utilization of less expensive metal alloys for fabricating implant-supported frameworks. In the present systematic review, only two studies compared cast frameworks constructed from gold alloy to other metal alloys.

Murphy et al. [28] investigated the clinical behavior of screw-retained frameworks fabricated in gold or silver-palladium (both veneered with acrylic resin). According to this study, both the two investigated materials had comparable accuracy of fit, resistance to functional stress and similar clinical outcomes, despite differences in mechanical properties. However, the authors specified that for silver-palladium, experience with the laboratory casting technique is necessary, in order to improve accuracy in this process. The most frequent technical complication for both the examined groups was: prosthetic screw fracture, abutment fracture, prostheses loosening, and artificial acrylic teeth fracture from frameworks. In any case, no correlation between framework material and clinical outcomes of the supporting implants could be found. For this reason, according to the authors, silver-palladium alloy may be considered a suitable lower-cost substitute for gold alloy for implant-supported frameworks.

Most of the other studies selected in the present review compared metal frameworks to more recent substitute materials, in order to evaluate if they could overcome some of the short- and long-term shortcomings of metal-acrylic (also called “hybrid”) prostheses (e.g., fracture of the acrylic resin veneer, prosthetic screw loosening/fracture, wear and fracture of resin denture teeth, fracture of prosthesis framework) [40].

Barootchi et al. [37] investigated technical complications in metal-acrylic and zirconia-based prostheses. Metal-acrylic prostheses showed a higher rate of minor complications (such as single tooth fracture or chipping, with *p* = 0.05), and a higher trend of delayed complications, even if it is worth specifying that the metal-acrylic group had a longer follow-up (9.5 years vs. 7 years for the zirconia prostheses).

The introduction of computer-aided design and computer-aided manufacturing (CAD/CAM) also allowed the entire design of the prostheses to be virtually managed and might obtain a superior fit compared to traditional metal frameworks [41].

However, some zirconia disadvantages, such as its high weight, as well as the difficult adjustment and polishing of the framework, still remain unsolved problems [37]. In addition, complications such as ceramic veneer chipping and the less frequent framework failure are reported.

According to some authors, a non-layered monolithic zirconia could solve the high incidence of ceramic chipping, by eliminating the presence of a zirconia/veneering ceramic interface [42,43]. The promising short-term clinical outcomes of monolithic zirconia complete-arch implant-supported prostheses are also supported by a systematic review by Abdulmajeed et al. [40]

In the present systematic review, Caramês et al. compared porcelain-veneered zirconia frameworks (PVZ) to monolithic zirconia with non-functional porcelain veneering (MZ). This study, in accordance with Barootchi et al., suggests that zirconia is a valuable material for full-arch frameworks, with low incidence of technical complications, high prosthodontic survival rate and implant success in the short/medium term. Caramês et al. also brought to light the improvement of mechanical properties of milled yttrium stabilized monolithic zirconia (3Y-TZPs). Although the difference was not statistically significant, the monolithic zirconia group presented lower rates of technical complications, which always occurred when the opposing arch was made of the same material. The study also recorded a higher peri-implant bone resorption in the PVZ group (mean 1 mm, *p* = 0.004).

Tartaglia et al. [33] compared CAD-CAM zirconia to CAD-CAM PMMA prostheses. According to the authors, the prosthesis material did not influence the risk of complications and failure, and fully veneered zirconia frameworks showed similar performance to resin ones, in terms of both technical and biological complications. As limits of this research, it must be acknowledged that the follow-up period was relatively short (up to 5 years) and that the authors fabricated zirconia frameworks instead of resin ones for all the 16 patients experiencing a fracture of the interim acrylic prosthesis. Despite such limitations, the high full-acrylic framework performances could be explained by the innovative fabrication process. In fact, the utilization of milling CAD-CAM techniques offers many advantages over the use of the traditional acrylic resin. Milled PMMA provides better mechanical properties compared to autopolymerizing acrylic resin, thanks to the lack of polymerization shrinkage. Finally, PMMA blocks are subjected to a process of polymerization under high pressure before milling, and this reduces the amount of residual monomer, enhancing hardness and wear resistance, and decreasing surface worsening and plaque accumulation on the material surface [44,45].

However, both traditional full-acrylic and PMMA frameworks can provide nowhere near the same mechanical properties, in terms of stiffness and rigidity, as metal frameworks. For this reason, many authors today consider full-resin a feasible material for long-term provisional implant-supported prostheses. Or else, it would be necessary to increase the prosthesis thickness, in order to achieve sufficient stiffness, and this is possible only if an adequate prosthodontic volume is present, or by remodeling the supporting bone [46].

Both the studies by Crespi et al. [31] and Tartaglia et al. [33] evaluated full-resin full-arch implant-supported prostheses but they had a too short follow-up to validate its use as a definitive material. In particular, Crespi et al. [31] found similar clinical outcomes comparing metal-acrylic and full-acrylic full-arch prostheses, with no fractures of prosthesis framework reported. However, two full-acrylic prostheses displayed fracture of the resin acrylic material. In addition, Crespi et al. [31] made no reference to the prosthodontic space available in their selected patients. The respect for the minimum thickness necessary for each specific material is fundamental for the long-term success of the rehabilitation. Tartaglia, at al. [33] in their study specified that the framework core was designed considering the veneering material, with additional caution for the zirconia framework group (in particular, unsupported porcelain could be 2 mm thick at the most, while the connectors within the crowns were designed with a 10 mm^2^ area at minimum). Most of the studies included in the present review did not specify this kind of detail, nor the shape of the framework. Therefore, it was not possible to understand if the veneering material was properly supported by the underlying substructure. Further factors not acknowledged by the majority of the studies but that might have affected the technical failures include: presence of cantilevered extensions, length of the prosthetic spans, occlusal scheme, type of antagonist, parafunctions, etc.

Titanium frameworks are also commonly used as alternatives to traditional castings, thanks to titanium’s biocompatibility, good resistance to corrosion and high mechanical properties. In addition, it has been suggested to provide a better passive fit to the implants, and this might be specific to the laser-welded titanium technique, because the precision of laser energy should minimize thermal expansion and contraction [47]. A passive fit would prevent loose screws and consequential fractures in prosthetics components. In the present research, four studies have compared different techniques for titanium framework fabrication to cast gold alloy substructures. However, among the studies included herein, Cannizzaro et al. [34] recorded significantly more complications for laser-welded titanium frameworks when compared to cast silver-palladium, with the rate of complications increasing after the third year of function. The authors suggested that laser-welded titanium frameworks should be chosen only for long-term temporary, and not definitive, prostheses. It must be noted that the study by Cannizzaro et al. described the use of two implants only in the mandible for full-arch immediate loading rehabilitations including distal cantilevers up to 1.5 cm long. This kind of rehabilitation may induce high mechanical stresses on the prosthodontic components, on the implants and on the peri-implant bone. As acknowledged by the authors themselves, “this therapeutic approach is experimental and not sufficiently validated”.

Similarly, Ortorp et al. [30] analyzed two different early generations of laser-welded implant-supported prostheses (Ti-1 and Ti-2 group) that were then reported together as a single test group (Ti-group). Ti-1 included a standard titanium bar framework consisting of titanium cylinders welded to titanium bars. The Ti-2 group included different pieces of titanium components with cylinders, which were placed on the master cast and then ground to the same level. To this flat plane, a titanium bar was laser-welded to complete the framework. The authors found that the 15-year prosthesis CSR was significantly better for cast gold alloy, in comparison to the first generation of titanium (Ti-1) frameworks (*p* = 0.041) which also registered a higher incidence of framework fractures (close to the terminal implant) (*p* = 0.034). Framework fracture was the most frequent mechanical complication together with resin veneer fractures. According to Ortorp et al. [30], framework and resin veneer fractures have been related to limited experience with laser-welding technique, similarly to what happened for cast metal frameworks at their first introduction. However, less mean bone loss for titanium frameworks was recorded, when compared to gold ones (*p* = 0.27).

Accordingly, Bergendal and Palmquist [27] found that the titanium group presented fractures of resin prosthetic teeth as a frequent complication. However, implant loss, framework fractures, passive fit, and marginal bone loss in titanium frameworks were not significantly different compared to cast-alloy frameworks.

Additionally, Jemt at al. [28] found that fracture of resin veneers was the most frequent mechanical problem; however, there were no statistical differences in the two compared groups. In addition, the authors did not record any fractures of implants, abutments or gold screws. In this study, marginal bone loss was comparable.

Laser-welding could be considered a weak link, and in order to make up for this disadvantage, Computer Numerical Controlled (CNC) milling techniques to fabricate one-piece titanium frameworks were introduced. These procedures may provide a better control of distortion, compared to conventional casting fabrication.

The present review took into consideration a study by Ortorp and Jemt [31] on CNC milled grade 2 titanium frameworks. This study reported two fractures of framework for both titanium and gold-alloy framework groups. Fractures of resin veneers were, again, one of the most common complications, and occurred more frequently in the early experienced one-piece milled technique. However, it must be noted that this study included patients treated between 1996 and 1998. The materials and fabrication techniques are now significantly improved and better outcomes are to be expected nowadays. In addition, in this study, few mechanical problems were recorded for the implant components. Similarly to Jemt et al., no differences in bone levels and bone loss were recorded.

Dealing with biological complications, only seven of the included studies [27,28,29,30,32,34,37] made specific reference to this topic. In addition, even when this estimation was present, it should be taken with caution, because of the variability in the rehabilitations, the different follow-up time, the study design and the not uniform definition of peri-implantitis among the different studies. The biocompatibility of non-noble metal alloys has been questioned. According to a recent systematic review, it is well known that metals can go into corrosion, and consequently induce local and systemic effects, or hypersensitivity reactions can appear [48].

Murphy et al. [29] reported that four patients in the gold alloy group and six in the silver-palladium alloy group experienced severe periabutment disease. Jemt et al. [28] found a tendency toward more soft tissue problems (such as inflammation or fistula) in the conventional cast-gold alloy framework group in comparison to the laser-welded group.

Compared to cast metal frameworks, titanium fabrication techniques allow for a potentially lower risk of oral corrosion, resulting in a more biocompatible device. [49] Ortorp et al. [30] underlined the biocompatibility and the low allergic potentials of titanium; however, they found that soft tissue inflammation was more common in the Ti-2 group in comparison to the gold alloy group during 0 to 15 years (*p* = 0.032).

In the literature [50], the onset of peri-implantitis in the case of metal-acrylic resin full-arch implant-supported prostheses has been sometimes related to the acrylic-resin veneer close to peri-implant soft tissues. Its porosity and progressive wear might increase plaque accumulation, with consequent difficulty for the patients in maintaining a proper home oral hygiene.

Among the studies comparing zirconia to cast metal frameworks, Barootchi et al. [36] recorded a similar prevalence of biological complications related to the prosthesis between both groups, despite the well-known higher biocompatibility and lower plaque accumulation of zirconia. They found denture-induced soft tissue complications, such as hyperplasia, prosthesis-induced ulcerations, pain and soreness induced by the acrylic, candidiasis, and gingival overgrowth. A higher (but not significant) percentage of cases of peri-implantitis (24% against 18.6%) was recorded in the zirconia group.

It is also generally assumed that a more precise passive fit of prosthetic screw-retained frameworks leads to a lower incidence of biological and technical complications. In this regard, successful techniques to passivate cast metal frameworks (such as the luting technique used to lute implant cylinders to metal frameworks [51,52]) can be used. However, there is still limited clinical evidence that misfitting directly causes any adverse biological complication. [11]

The study by Pera et al. [35] was the only one investigating a framework made of carbon fiber-reinforced composite (CFRC). They found greater implant survival and less bone resorption in patients rehabilitated with CFRC frameworks compared to cast metal frameworks. No prosthetic complications occurred during the follow-up period (mean: 22 months) of CFRC frameworks. In addition to high rigidity, resistance and tenacity (comparable to those of metal frameworks), CFRC has an excellent fatigue resistance, and good shock absorption and energy dissipation capacity. Finally, it provides a chemical adhesion to the veneering acrylic resin: this is expected to reduce the occurrence of chipping of the veneering material, which is a common technical complication in implant prosthodontics [7]. In addition, CFRC prostheses are cheap, easy-to-produce (avoidance of casting and no need of costly machineries or instruments for the manufacturing, and no need for post-passivation), and lightweight [25]. This latter point might be crucial for patients’ comfort, besides the fact that according to a recent FEM analysis by Tribst et al., heavier prostheses under the effect of gravity force are related to more strain being generated around the implants. However, in contrast with metal alloy, CFRC is an anisotropic and non-homogeneous material, due to the fact that it is created by superimposing layers of carbon fibers embedded in a polymer matrix. For this reason, the manufacturing technique strongly affects the final characteristics of the prostheses [53]. A preliminary in vitro study on carbon fiber frameworks for dental implant applications [7] also reported that intact and fragmented carbon fiber samples showed optimal biocompatibility.

The present research has some potential limitations. The first one derives from information lacking in some of the included studies. For example, not all the studies simultaneously reported all the outcomes investigated in the present review. In addition, the so called “technical” and “biological complications” that were reported included a wide range of clinical problems encountered, often different from one study to another.

Finally, the studies included show heterogeneity in their follow-up periods, from a minimum of 1 year up to a maximum of 15 years follow-up. More caution should be taken interpreting the results of the studies with shorter follow-up.

In addition, some less common materials that are nowadays proposed for framework realization, such as high performance polymers (HPP), were not taken into consideration in the present review since no papers were found meeting the inclusion criteria.

In conclusion, the present systematic review suggests that conventional cast noble (gold or silver-palladium) or not noble metal alloys (Co-Cr) are the most traditionally employed materials for full-arch implant-supported rehabilitations, thanks to their great mechanical properties and a proven technique, which guarantees high clinical performances with optimal clinical implant and prosthetic survival rates in the long term. However, various alternative materials are available today, such as titanium, zirconia and several polymers including carbon-fiber frameworks, providing corrosion resistance and biocompatibility, great mechanical characteristics, with satisfactory clinical outcomes. In addition, when a CAD/CAM fabrication process is employed, less dependence on manual laboratory procedures is provided, a better fit between framework and dental implants may be achieved, and a completely digital workflow can be applied. However, prosthesis provided with these new materials are not free of technical and biological complications. It must also be underlined that the clinical outcomes of the rehabilitation are strongly affected not only by the type of material employed but also by the design of the prosthesis and its manufacturing technique. For these reasons, the development of standardized protocols and a learning curve in manufacturing is recommended.

Further comparative clinical studies, possibly randomized clinical trials with a longer follow-up-time, are needed in order to validate the use of new materials and define their specific clinical indications.

## Figures and Tables

**Table 1:** Main characteristics and outcomes of the included studies.

Authors (Year)	Study Type	Type of Loading	N° of Implants	N° of Patients	Test	Control	Jaw	Follow-Up	Results	Conclusions
Bergendal and Palmquist 1999 [27].	Multicenter prospective cohort study, with a historical control group	Not reported	818: 415 (test) 403 (control)	184 (reported until the 2-years follow-up)	Laser-welded titanium framework; 67 at the 5-years examination	Gold-alloy framework; 66 at the 5-years examination	Maxilla and mandible	2 and 5 years	− Mechanical complications: abutment screw fracture, gold screw fracture, fracture of artificial teeth and/or acrylic resin material were, respectively, 0, 1, 17, 8 (test) and 1, 0, 7, 2 (control); more fractures of framework and of artificial teeth occurred in the test group (no statistical difference). − Biological complications: higher incidence of soft tissue problems in test group. (8 vs. 2 cases) − pCSR: not reported − iCSR: 98.6% (test) and 99% (control); no statistical difference − BR: no statistical differences	Titanium framework clinical behavior is encouraging
Jemt et al. 2002 [28].	Multicenter randomized clinical trial	Not reported	349	58: 28 (test) vs. 30(control)	Laser- welded titanium	Cast-gold alloy	Maxillary	5 years	− Technical complications: few mechanical problems (fracture of resin veneers was the most frequent); no fracture of metal components; 4 (test) and 2 (control) frameworks presented loose gold screws; − Biological complications: a tendency toward more soft tissue problems was reported in control group. − iCSR: 91.4% (test) and 94.0% (control) from implant insertion (no statistical difference) − *p*-CSR 96.4% and 93.3%.; *p* = 0.002 − BR: on average 0.59 mm (SD 0.97 mm); no statistical difference.	No clinical or radiographic differences were observed between the two groups (similar favorable clinical performance).
Murphy et al. 2002 [29].	Prospective	Delayed (3 months)	Not directly reported. 66–67 implants (test) vs. 65–66 (control) can be deducted from the text	26	Gold alloy	Silver-palladium alloy	Mandibular	5 years	− Technical complications: 1 prosthesis screw fractured in each group; 1 abutment fractured in control group; 11 (test) and 4 (control) loose prostheses; 2 artificial teeth fractured from frameworks in each group. − Biological complications: 4 (test) and 6 (control) patients had severe periabutment disease. − iCSR: 92% (test), 95% (control); 3 (test) and 1 (control) implants were not used for loading. − pCSR − BR: no statistical difference; the majority of patients had bone changes less than 1 mm.	Despite differences in mechanical properties, clinical performance of both materials and radiographic changes in peri-implant bone was similar over 5 years (similar accuracy of fit and resistance to functional stress). Therefore, silver-palladium alloy may be considered a suitable low-cost substitute for gold alloy for implant- supported frameworks.
Ortorp et al., 2009 [30]	Post-trial follow-up	Delayed	821	155 (test) vs. 53 (control); 53 (test) vs. 13 (control) reached the 15-years follow-up	Laser-welded titanium	Gold alloy castings	Mandibular	15 years	− Technical or biological complications: the most − common complication for test group was resin or veneer/fillings fractures and soft tissue inflammation. Fractures in test group occurred more frequently (15.5%, *p* =.034). Loose and fractured implant. Screw components were few (2.4%). − pCSR: 89.2 (test) and 100% (control) (*p* = 0.057) (overall CSR 91.7%). − iCSR: 98.7%. The 72 patients still had a fixed prosthesis at the termination of the study. − BR: 0.59 mm (test) and 0.98 mm (control); *p* = 0.027. − However, there is still a low clinical significance related to this difference in the present study. − Few (1.3%) implants had >3.1 mm bone loss after 15 years.	The gold alloy frameworks had a tendency to work better when compared with welded titanium frameworks over 15 years. However, more bone loss was observed for implants supporting gold alloy frameworks.
Crespi et al. 2012 [31].	Randomized clinical trial	Immediate	176	36	Full-acrylic	Metal-acrylic	Maxillary and mandibular	3 years	− Technical complications: no fractures of frameworks in any groups. − Biological complications: not reported − pCSR: 100% but 2 all-acrylic resin prostheses displayed fractures of the acrylic resin material − iCSR: overall iCSR 100% for axial implants and 96.59% − for tilted implants. iCSR 98.96% in the maxillary; 97.5% in the mandible − BR: 1.10 ± 0.45 mm axial implants; 1.11 ± 0.32 mm tilted implants in the maxillary); 1.06 ÷ 0.41 mm axial implants; 1.12 ± 0.35 mm tilted implants in the mandible. No statistical difference	The same clinical outcomes were found, regardless of whether the acrylic-resin restorations were reinforced with metal
Ortorp et al. 2012 [32].	Randomized clinical trial	Delayed	728	126: 65 (test) vs. 61 (control) 36 (test) 38 (control) reached the 10-years follow-up	Computer Numerical Controlled (CNC) titanium	Cast gold-alloy	Maxillary and mandibular	10 years	− Technical complications: 1 prosthesis lost in each group due to loss of implants; 1 prosthesis failed due to − framework fracture in the test group. 2 metal fractures were registered in each group. The frequency of complications was low − Biological complications: no signs of biological different response to titanium frameworks have been observed − pCSR: 95.6% (test) 98.3% (control); *p* > 0.05; 1 prosthesis failed due to framework fracture in the test group. − iCSR: 95.0% (test) 97.9% (control); *p* > 0.05; no implants were lost after 5 years. − BR: in test group 0.7 mm (maxillae) and 0.7 mm (mandible); no statistical difference with the control group, (*p* > 0.05) with similar clinical and radiological performance for both groups over 10 years.	CNC-milled titanium frameworks are a viable alternative to gold-alloy castings for restoring patients with implant-supported protheses in the edentulous jaw.
Tartaglia et al. 2015 [33].	Retrospective cohort study	Immediate	1058	113	CAD-CAM zirconia prostheses veneered with feldspathic porcelain*	CAD-CAM PMMA prostheses veneered using composite resin teeth*	Maxilla and mandible	2–60 months	− Technical complications: no statistical difference; prostheses annual complication rate 6.6% vs. free complications prosthetic survival rate 75.5 %; prostheses annual failure rate 4.6% vs. free survival 85.5 %; no statistical difference − Biological complications: not reported − BR: not reported	Prosthesis material did not influence complication risk.
Cannizzaro et al. 2016 [34].	Prospective cohort study	Immediate	160	80	Laser- welded titanium	Cast silver- palladium	Mandibular	5 years	− Biomechanical or biological complications: significantly more complications (19 patients out of 46 and 6 patients out of 34, respectively), *p*= 0.032 in test group. − pCSR: 10 prostheses were remade. − iCSR: 2 implants failed early in two patients, but they were successfully replaced. Mean ISQ values decreased from 75.4 to 73.8. − BR: 0.69 mm	Laser-welded framework constructionshould be considered as a long-term temporary prosthesis and not definitive. Immediately loaded mandibular cross-arch prostheses can be supported by only two implants up to 5 years, if made with a robust cast framework.
Pera et al. 2017 [35].	Prospective cohort study, with a historical control group	Immediate	333: 170 test) vs. 163 (control)	76: 42 (test) vs. 34 (control)	Carbon fiber frameworks	Cast metal framework (34 patients163 implants)	Maxilla	22 months (range: 18–24)	− Technical complications: not reported − Biological complications: not reported − pCSR: not reported − iCSR: 100% (test) vs. 93.9% (control); *p* = 0.002-BR: higher in control group (mean 1 mm, *p*= 0.004)	Carbon fiberframeworks may be considered as a viablealternative to the metal ones and showed lessmarginal peri-implant bone loss and a greaterimplant survival rate.
Caramês et al. 2019 [36].	Prospective cohort study	Delayed: after 12 weeks or after 6–9 months (in case of bone regeneration); during healing and osseointegration immediate provisional restoration consisted in metal-reinforced fixed complete dentures	1009: 581 (test) vs. 428 (control)	132: 62 (test) vs. 70 (control)	MZ (milled Yttrium-stabilized monolithic zirconia) with veneering porcelain limited to non-functional surfaces	PVZ (feldspathic porcelain-veneered zirconia)	Maxilla and mandible	From 1 to 2 years	− Technical complications: low incidence for both groups (total complication rate 11.3%); the most prevalent complications were loss of the access chamber composite plug and prosthetic screw loosening. Minor, major chipping, framework fracture or any of the former combined to occur was 0.99, 0.95, 0.95, 0.89 and (test) 0.99, 0.95, 0.93 and 0.89 (test); no statistical difference − Biological complications: not reported − pCSR: 99.0% (test) vs. 98.7% (control); no statistical difference − iCSR: implant success 99.83% (test) vs. 99.53%(control); no statistical difference − BR: not reported	Zirconia (in particular MZ group) has demonstrated to be a suitable material for frameworks in full-archImplant-supported rehabilitations.
Barootchi et al. 2020 [37].	Retrospective cohort study	Immediate or delayed	452: 200 (test) vs. 252 (control)	56: 35 (test) vs. 21 (control)	Monolithic zirconia framework with luted single ceramic crowns and light cured resin to mimic gingival tissue	Cast metal-acrylic	Maxilla and mandible	≥ 5 years (7 test; 9,5 control)	− Technical complications: early c. no statistical difference; delayed c. no statistical difference but a higher trend in control group (*p* = 0.074); minor c. (single-tooth fracture/dislodgment/chipping) more frequent in both groups but higher in control group (*p* = 0.05). − Biological complications: similar. − pCSR: 93.7% ± 5% (test) vs. 83.0% ± 11.1% (control) (*p* = 0.46) − iCSR: implant failure 19.4% (test) vs. 23.3% (control). No statistical difference. − BR: not reported	Zirconia fixed implant prostheses presented higher initial costs than metal-acrylic hybrids, but with satisfactory outcomes, reduction of overall complications, and superior survival rates

* In both test and control group, the immediate loading prosthesis was a 10-tooth acrylic screw-retained interim prosthesis. After 2 months, the final prosthesis material was selected.

**Table 2:** Risk of bias for the randomized clinical trials included in the present systematic review according to Cochrane Handbook for Systematic Reviews of Interventions.

Study	Selection Bias Sequence Generation	Selection Bias Allocation Concealment	Performance Bias	Detection Bias	Attrition Bias	Selective Reporting Bias	Other Potential Risk of Bias
Jemt et al. 2002	Low	Low	Unclear	Unclear	Low	Low	Low
Ortorp and Jemt. 2009	High	High	Unclear	Unclear	Low	Low	Low
Crespi et al. 2012	Unclear	Unclear	Unclear	Unclear	High	Unclear	Low
Ortorp and Jemt. 2012	Unclear	Unclear	Unclear	Unclear	Low	Low	Low

**Table 3:** Risk of bias for the cohort studies included in the present systematic review according to the NOS-Newcastle–Ottawa Scale. A star system was used in order to perform a semi quantitative assessment of study quality. A study was awarded a maximum of one star ("*") for each numbered item satisfied in Selection, Comparability and Exposure/outcome categories. The symbol "-" means that the item was not satisfied.

Study	Selection	Comparability	Outcome/Exposure	NOS Score
Bergendal and Palmquist 1999	--✶✶	✶-	✶✶-	5
Murphy et al. 2002	--✶✶	✶-	✶✶-	5
Tartaglia et al. 2015	✶✶✶✶	✶-	✶✶-	7
Cannizzaro et al. 2016	✶✶✶✶	✶-	✶✶-	7
Pera et al. 2017	✶✶✶✶	--	✶✶-	6
Caramês et al. 2019	✶✶✶✶	✶✶	✶✶✶	9
Barootchi et al. 2020	✶✶✶✶	--	✶✶✶	7

## Data Availability

Data sharing not applicable.

## Appendix A

**Table A1:** Table reporting the 11 excluded studies and reasons for exclusion.

Study	Reason for Exclusion
Jemt et al. 1998	(redundant)
Bergendal and Palmquist 1995	(redundant)
Ortorp et al. 2000	(redundant)
Ortorp et al. 2002	(redundant)
Ortorp et al. 2004	(redundant)
Cannizzaro et al. 2014	(redundant)
Pozzi et al. 2015	(non-comparative)
Merli et al. 2017	(insufficient follow-up)
Wolff et al. 2018	(non-comparative)
Castorina et al. 2019	(case report)
Hulterström et al. 1991	(only one framework material tested)
